# ERG-associated protein with SET domain (ESET)-Oct4 interaction regulates pluripotency and represses the trophectoderm lineage

**DOI:** 10.1186/1756-8935-2-12

**Published:** 2009-10-07

**Authors:** Leng-Siew Yeap, Katsuhiko Hayashi, M Azim Surani

**Affiliations:** 1 Wellcome Trust/Cancer Research UK Gurdon Institute, University of Cambridge, Cambridge, UK; 2 Department of Anatomy and Cell Biology, Graduate School of Medicine, Kyoto University, Yoshida-Konoe-Cho, Sako-Ku, Kyoto 606-8501, Japan

## Abstract

**Background:**

Pluripotency, the capacity for indefinite self-renewal and differentiation into diverse cell types is a unique state exhibited by embryonic stem (ES) cells. Transcriptional regulators, such as Oct4, are critical for pluripotency, but the role of epigenetic modifiers remains to be fully elucidated.

**Results:**

Here, we show that ERG-associated protein with SET domain (ESET), a histone methyltransferase enzyme, maintains pluripotency through repression of *Cdx2*, a key trophectoderm determinant, by histone H3 lysine 9 trimethylation (H3K9me3) of the promoter region. Notably, this repression is mediated through the synergistic function of small ubiquitin-related modifier (SUMO)ylated ESET and Oct4. ESET localises to the promyelocytic leukaemia (PML) nuclear bodies and is SUMOylated in ES cells. Interaction of ESET with Oct4 depends on a SUMO-interacting motif (SIM) in Oct4, which is critical for the repression of *Cdx2*.

**Conclusion:**

Loss of ESET or Oct4 results in strikingly similar phenotypes both in ES cells with their differentiation into trophectoderm cells, and in early embryos where there is a failure of development of the pluripotent inner cell mass (ICM) of blastocysts. We propose that SUMOylated ESET-Oct4 complex is critical for both the initiation and maintenance of pluripotency through repression of differentiation, particularly of the trophectoderm lineage by epigenetic silencing of *Cdx2*.

## Background

ERG-associated protein with SET domain (ESET), also known as SET domain bifurcated 1 (SETDB1), is a histone methyltransferase that catalyses a repressive mark on euchromatin by mediating histone 3 lysine 9 trimethylation (H3K9me3) [1,2]. The ESET protein contains a Tudor domain, a methyl-CpG binding domain and a bifurcated SET domain that is responsible for its catalytic activity [3]. Proteins that associate with ESET are mainly corepressors [1,4-7], in agreement with its repressive role. ESET is critical for very early development since the *Eset*-null embryos die at the peri-implantation stage with defective development of the inner cell mass (ICM), from which no embryonic stem (ES) cells could be derived [8]. The *Eset-*null phenotype is similar to that of *Oct4*-null embryos [9] but the basis for this is unknown. We decided to address this question by investigating the role of ESET in the ICM-derived pluripotent ES cells.

## Results and Discussion

### ESET-depleted ES cells differentiate towards the trophectoderm lineage

**Figure 1:**
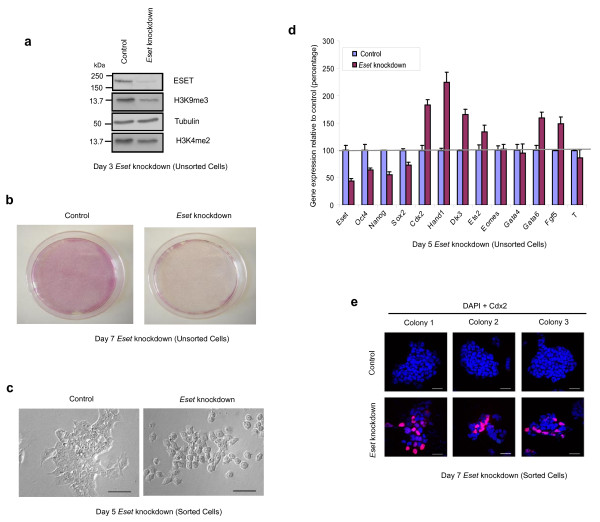
**ERG-associated protein with SET domain (ESET)-depleted embryonic stem (ES) cells differentiate towards the trophectoderm lineage**. **(a) **Western blot shows downregulation of ESET and histone 3 lysine 9 trimethylation (H3K9me3) in *Eset *knockdown ES cells at day 3 of short hairpin RNA (shRNA) transfection. Tubulin and H3K4me2 served as loading control. **(b) **Alkaline phosphatase staining of *Eset *knockdown (right panel) and control (left panel) ES cells after 7 days of shRNA transfection. **(c) **Morphology of fluorescence-activated cell sorting (FACS)-sorted *Eset *knockdown (right panel) and control (left panel) ES cells at day 5 of shRNA transfection. Scale bar, 50 μm. **(d) **Gene expression levels in *Eset *knockdown ES cells relative to control cells after normalising against *Gapdh*, a house keeping gene. Error bars, standard deviation (SD) of three technical replicates. **(e) **Images of three representative colonies from three different wells of control (top panel) and *Eset *knockdown (bottom) ES cells which were purified by FACS at day 3 of shRNA transfection followed by 4 days of culture in medium that is conducive for development of trophectoderm cells (TS medium). Cdx2-positive cells are labelled in red. Nuclei are labelled in blue. Scale bar, 100 μm.

To investigate the role of ESET in ES cells, we induced knockdown of *Eset *using a short hairpin RNA (shRNA)-expressing vector that contains an enhanced green fluorescent protein (EGFP) reporter (Figure 1a). An empty vector was used as a control. At 7 days following transfection of *Eset *shRNA, we observed a dramatic loss of pluripotent ES cells as judged by a marked reduction in the number of alkaline phosphatase positive cells (Figure 1b, Additional file 1). To visualise the morphology of the knockdown cells, EGFP-positive cells were purified by fluorescent activated cell sorting (FACS), 24 h after transfection and cultured in ES medium for 2 to 4 days (Figure 1c, Additional file 1). Notably, ES cells transfected with *Eset *shRNA appeared flat and differentiated, which was clearly distinct from control ES cells, which formed compact colonies (Figure 1c). This experiment demonstrates that ESET is important for the maintenance of undifferentiated ES phenotype.

Next, 5 days after transfection of *Eset *shRNA, we carried out reverse transcription quantitative polymerase chain reaction (Q-PCR) analysis to determine the precise consequences of *Eset *knockdown. Concomitant with the change in the phenotype in ESET-depleted ES cells, we found downregulation of pluripotency-specific genes, including *Oct4*, *Nanog *and *Sox2*. At the same time, we detected upregulation of genes associated with differentiation, including, *Cdx2*, *Hand1*, *Dlx3*, *Ets2*, *Fgf5 *and *Gata6 *(Figure 1d). Notably, this response is similar to that triggered by the knockdown of *Oct4 *in ES cells, where genes associated with the trophectoderm lineage, such as *Cdx2 *and the primitive endoderm lineage, such as *Gata6 *are upregulated [10]. Thus, apart from the failure of ICM development and pluripotent cells in early embryos following the loss of either *Eset *[8] or *Oct4 *[9], the response of ES cells to the loss of these two genes is also comparable.

The upregulation of trophectoderm-specific genes is of particular interest as mouse ES cells have a low propensity to differentiate into trophectoderm cells [11]. This is, at least in part, because Oct4 and Cdx2 induce reciprocal inhibition to achieve a mutually exclusive expression in the pluripotent and the trophectoderm lineages, respectively [12]. Since *Eset *knockdown evidently exhibits a similar outcome as *Oct4 *knockdown in ES cells, we investigated whether ESET, like Oct4, might also play a role in repressing trophectoderm differentiation. We tested this possibility by investigating whether ESET-depleted ES cells could give rise to trophectoderm cells. To do this, we cultured ESET-depleted ES cells in a medium that is conducive for development of trophectoderm cells (henceforth called TS medium) [13]. Notably, Cdx2-positive cells were observed in about 40% of *Eset *knockdown cells after 4 days of culture in TS medium (Figure 1e). By contrast, the majority of EGFP-negative cells cultured in the TS medium, which represents a concurrent control, were negative for Cdx2, except for a few cells that are occasionally positive (less than 1% of total cells), which arise spontaneously when ES cells are exposed to TS medium. The heterogeneity of expression of Cdx2 in ESET-depleted ES cells suggests that, apart from the trophectoderm cells, these ESET-depleted cells may differentiate into other lineages. Nevertheless, ESET-depleted ES cells clearly have a propensity to differentiate towards the trophectoderm lineage, a response that is uncharacteristic of normally differentiating ES cells. To gain mechanistic insight on the role of *Eset *in regulating pluripotency of ES cells, we decided to focus our analysis on *Cdx2*, since this is an early and a key determinant of the trophectoderm lineage [14].

### ESET-mediated H3K9me3 represses *Cdx2 *in ES cells

**Figure 2:**
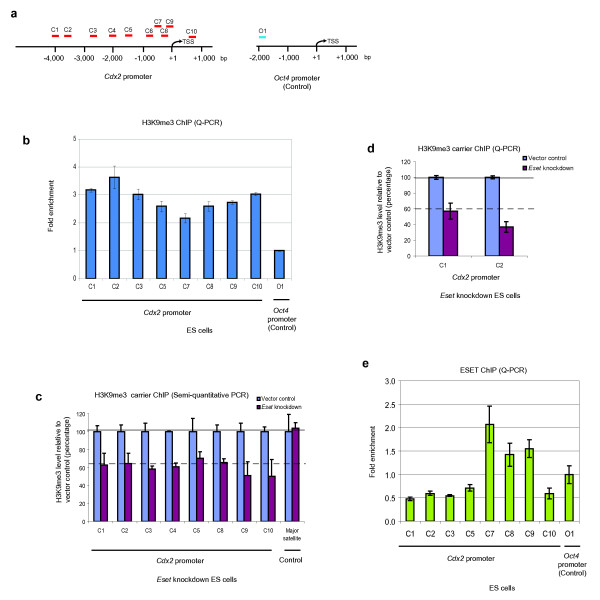
**ERG-associated protein with SET domain (ESET)-mediated histone 3 lysine 9 trimethylation (H3K9me3) represses *Cdx2 *in embryonic stem (ES) cells**. **(a) **Chromatin immunoprecipitation (ChIP) primers C1 to C10 used to detect enrichment of H3K9me3 on *Cdx2 *promoter (left). Primers O1 of *Oct4 *promoter served as a negative control (right). The numbers below the bars indicate distance from transcription start site (TSS) in base pairs (bp). **(b) **Quantitative polymerase chain reaction (Q-PCR) analysis of the enrichment of H3K9me3 at different positions along *Cdx2 *promoter region relative to the *Oct4 *promoter region after normalising against H3 ChIP and IgG controls. Primers C4 and C6 were not suitable for Q-PCR analysis. Error bars, standard deviation (SD) of three technical replicates. **(c) **Carrier ChIP semiquantitative PCR analysis of H3K9me3 at the *Cdx2 *promoter and major satellite regions of fluorescence-activated cell sorting (FACS)-sorted *Eset *knockdown ES cells relative to control cells after normalising against their respective input and IgG controls. Primers C6 and C7 were not suitable for carrier ChIP analysis. Error bars, SD of three independent experiments. **(d) **Q-PCR analysis of the levels of H3K9me3 at region C1 and C2 of the *Cdx2 *promoter in *Eset *knockdown ES cells relative to control cells after normalising against their respective input and IgG controls. Error bars, SD of three independent experiments. **(e) **Q-PCR analysis of the enrichment of ESET on different positions along the *Cdx2 *promoter relative to the *Oct4 *promoter after normalising against their respective input and IgG controls. Error bars, SD of three technical replicates.

Since the loss of *Eset *from ES cells results in the upregulation of *Cdx2 *at the mRNA and protein levels, we reasoned that *Cdx2 *might be repressed by ESET-mediated epigenetic modification at its promoter region via H3K9me3. To test for this possibility, we first performed chromatin immunoprecipitation (ChIP) for H3K9me3 at the *Cdx2 *promoter region in wild-type ES cells. We detected H3K9me3 marks that were spread throughout the 5 kb region surrounding the transcription start site of *Cdx2 *gene (Figure 2a, b, Additional file 2). In contrast, H3K9me3 levels were not enriched at the promoter region of *Oct4 *gene, which unlike *Cdx2 *is transcriptionally active in ES cells (Figure 2a, b).

To investigate whether ESET is the histone methyltransferase responsible for H3K9me3 modification at the *Cdx2 *promoter region, we performed ChIP analysis on ES cells. H3K9me3 was detected at the *Cdx2 *promoter region, but this signal was downregulated in ESET-depleted cells by an average of 40% to 50% compared to control cells (Figure 2c, Additional file 2). This observation was confirmed by Q-PCR analysis using primers C1 and C2 (Figure 2d). As an additional control, we also analysed the H3K9me3 at the major satellite region that is known to be mediated by Suv39h1/2, a histone methyltransferase that governs H3K9me3 at the pericentric heterochromatin region [15,16]. In contrast to the observations on the *Cdx2 *promoter region, H3K9me3 levels at the major satellite region were unaffected in ESET-depleted ES cells (Figure 2c, Additional file 2). These results suggest that the H3K9me3 marks at the *Cdx2 *promoter region were most likely mediated by ESET.

To further confirm that ESET mediates H3K9me3 at the *Cdx2 *promoter region in ES cells, we performed ChIP using an anti-ESET antibody. We detected enrichment of ESET on *Cdx2 *promoter with a peak at the region immediately upstream of the transcription start site, which was amplified by primer C7 (Figure 2e). Collectively, these results demonstrate that ESET-mediated H3K9me3 represses *Cdx2 *in ES cells.

### Oct4 and ESET synergistically repress *Cdx2*

**Figure 3:**
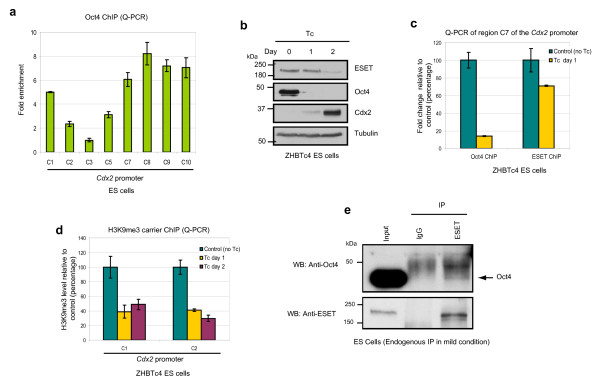
**Oct4 and ERG-associated protein with SET domain (ESET) synergistically repress *Cdx2***. **(a) **Quantitative polymerase chain reaction (Q-PCR) analysis of the enrichment of Oct4 on different positions along the *Cdx2 *promoter relative to the least enriched region (C3) after normalising against their respective input and IgG controls. Error bars, standard deviation (SD) of three technical replicates. **(b) **Western blot shows upregulation of Cdx2 and downregulation of ESET upon depletion of Oct4 at day 2 of tetracycline (Tc) treatment of ZHBTc4 embryonic stem (ES) cells. **(c) **Q-PCR analysis of the levels of Oct4 and ESET enrichment at region C7 of the *Cdx2 *promoter in ZHBTc4 ES cells treated with Tc for 1 day relative to untreated cells after normalising against their respective input. Error bars, SD of three independent experiments. **(d) **Carrier chromatin immunoprecipitation (ChIP) Q-PCR analysis of the levels of histone 3 lysine 9 trimethylation (H3K9me3) at region C1 and C2 of the *Cdx2 *promoter in ZHBTc4 ES cells treated with Tc for the indicated days relative to untreated cells after normalising against their respective input and IgG controls. Error bars, SD of three independent experiments. **(e) **ES cell lysates were immunoprecipitated (IP) with anti-ESET antibody (kind gift of HH Ng; see text) under mild conditions in digitonin-containing buffer and subjected to western blotting (WB) with the antibodies indicated. Rabbit IgG was used as a negative control.

Next, we considered whether Oct4 and ESET act synergistically to repress *Cdx2 *since loss of function of *Eset *and *Oct4 *generate similar phenotypes, both during early embryonic development [8,9], and in ES cells, as described above. First, we confirmed that Oct4 is enriched on *Cdx2 *promoter in wild-type ES cells (Figure 3a) as reported previously [17]. Notably, Oct4 is enriched at the same region that is bound by ESET (Figure 2e), suggesting that both factors might coregulate *Cdx2*. To address this possibility, we used ZHBTc4 ES cells that contain a tetracycline regulatable *Oct4 *allele [18]. Depletion of Oct4 from these ES cells following addition of tetracycline to the culture medium results in the induction of Cdx2 after 2 days (Figure 3b). To analyse the effect of Oct4 depletion on the binding of ESET to *Cdx2 *promoter, we performed ChIP analysis on ZHBTc4 ES cells at day 1 of tetracycline treatment when ESET expression was not yet affected by the depletion of Oct4 (Figure 3b). As expected, Oct4 binding on the *Cdx2 *promoter was downregulated by 90% in Oct4-depleted ES cells (Figure 3c). Notably, this was accompanied by downregulation of ESET binding on the *Cdx2 *promoter by 30% (Figure 3c). Furthermore, downregulation of ESET binding was coupled with the decline of H3K9me3 levels at the *Cdx2 *promoter region (Figure 3d). We further verified that the binding of ESET to *Cdx2 *promoter is dependent on Oct4 by performing ChIP experiments in ZHBTc4 ES cells overexpressing haemagglutinin (HA)-tagged ESET (Additional file 3). In this experiment, tetracycline was first added to ZHBTc4 cells to initiate loss of Oct4, followed 1 day later by transfection of HA-tagged ESET, followed by a ChIP experiment 1 day further on using an anti-HA antibody. The results show that ESET binding to *Cdx2 *promoter decreased by 40% confirming that the loss of Oct4 affects the binding of ESET to *Cdx2 *promoter. Taken together, these results suggest that ESET and Oct4 likely cooperate to repress *Cdx2*, which is consistent with the observation that depletion of either ESET or Oct4 leads to upregulation of Cdx2.

**Figure 4:**
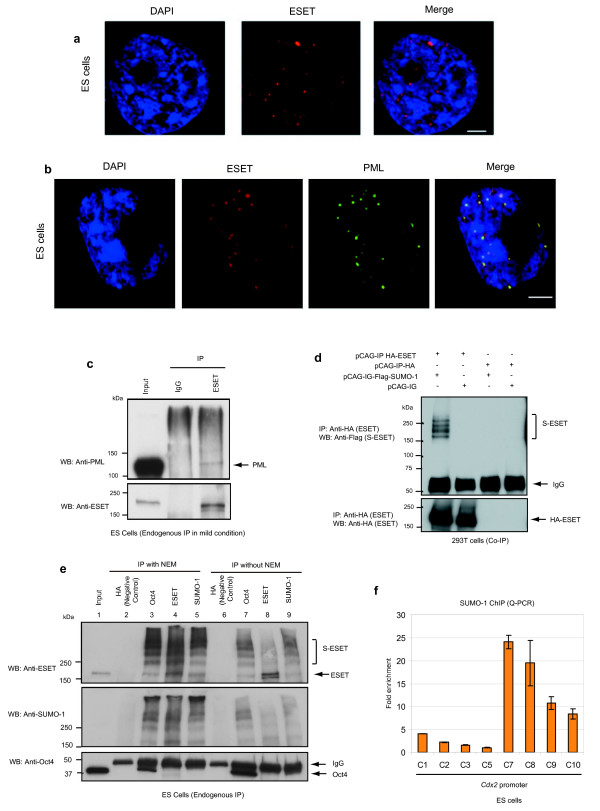
**ERG-associated protein with SET domain (ESET) localises to promyelocytic leukaemia (PML) nuclear bodies and is post-translationally modified by small ubiquitin-related modifier (SUMO)**. **(a) **ESET (red) localises to distinct, punctate foci in euchromatin regions, as indicated by the absence of DAPI (blue) staining in embryonic stem (ES) cells. Scale bar, 3 μm. **(b) **ESET (red) localises to distinct, punctate foci that overlaps with PML nuclear bodies (green) in ES cells. Nuclei are labelled in blue. Scale bar, 5 μm. **(c) **ES cell lysates were immunoprecipitated (IP) with anti-ESET antibody (kind gift of HH Ng; see text) under mild conditions in digitonin-containing buffer and subjected to western blotting (WB) with the antibodies indicated. Rabbit IgG was used as a negative control. **(d) **Western blot analysis of coimmunoprecipitation experiment in 293T cells transfected with haemagglutinin (HA)-ESET and/or Flag-SUMO-1. S-ESET represents SUMOylated ESET. **(e) **ES cell lysates were immunoprecipitated (IP) with the indicated antibodies in NP40-containing buffer either in the presence or absence of *N*-ethylmaleimide (NEM) and subjected to western blotting (WB) using 4% to 15% Tris-HCl gradient gel. A rabbit anti-HA antibody was used as negative control. **(f) **Quantitative polymerase chain reaction (Q-PCR) analysis of the enrichment of SUMO-1 on different positions along *Cdx2 *promoter relative to the least enriched region (C5) after normalising against their respective input and IgG controls. Error bars, standard deviation (SD) of three technical replicates.

Since *Cdx2 *is a known target of Oct4 [12,17,19], it is possible that Oct4 may interact with ESET to repress *Cdx2*. To test for this possibility, we coexpressed Oct4 and ESET in 293T cells and found that ESET coimmunoprecipitated with Oct4 (Additional file 3). To further investigate which domain of ESET interacts with Oct4, we generated two deletion constructs; HA-ESET-ΔSET and HA-ESET-ΔTudor (Additional file 3). Interestingly, when we coexpressed either HA-ESET-ΔSET or HA-ESET-ΔTudor with Oct4, both mutants were able to coimmunoprecipitate with Oct4 (Additional file 3), suggesting that ESET and Oct4 form a complex through bridging proteins, or that ESET associates with Oct4 through at least two different sites. We also performed immunoprecipitation of endogenous ESET in ES cells using an anti-ESET antibody and found that Oct4 was coimmunoprecipitated independently of DNA, albeit at a low level and only when immunoprecipitation was carried out under mild conditions (Figure 3e and Additional file 4). However, in the reciprocal immunoprecipitation experiment using an anti-Oct4 antibody, we noticed that immunoprecipitation of endogenous Oct4 not only precipitated the expected ESET protein of 180 kDa, but also ESET proteins of higher molecular weight, which may be post-translationally modified ESET proteins (Figure 4e, lane 7, top panel and Additional file 4). We therefore sought to investigate these modified ESET proteins.

### ESET localises to promyelocytic leukaemia (PML) nuclear bodies and is post-translationally modified by small ubiquitin-related modifier (SUMO)

We had noted that ESET has a very striking pattern of subcellular localisation in ES cells, where it is mainly found in punctate foci specifically within the euchromatin region as indicated by the absence of 4',6-diamidino-2-phenylindole (DAPI) staining (Figure 4a). Notably, we found that these ESET punctate foci overlap with PML nuclear bodies (Figure 4b). Coimmunoprecipitation experiments in 293T (Additional file 5) and ES cells under mild conditions (Figure 4c and Additional file 4) show that ESET interacts with PML. PML nuclear bodies are of interest because these are highly organised structural and functional domains that are composed of the PML protein and various other proteins that are involved in different biological functions [20,21]. Post-translational modification of PML through addition of SUMOs is pivotal for the formation of PML nuclear bodies [22,23], and many proteins found in the PML nuclear bodies are SUMOylated [24,25]. Since ESET colocalises to PML nuclear bodies and interacts with PML in ES cells, we investigated whether ESET undergoes SUMOylation.

To test this hypothesis, HA-ESET and Flag-SUMO-1 were cotransfected in 293T cells and cell lysates were subjected to immunoprecipitation in the presence of *N*-ethylmaleimide (NEM), an inhibitor of SUMO isopeptidases. When cell lysate was immunoprecipitated with anti-HA antibody (to immunoprecipitate ESET) and immunoblotted with anti-Flag antibody (to visualise SUMOylated ESET), we observed several slower migrating ESET proteins, which may be indicative of different degrees of SUMOylation of ESET protein (Figure 4d). This result suggests that ESET may be SUMOylated in ES cells and that the high molecular weight ESET proteins that interact with Oct4 are probably SUMOylated forms of ESET proteins.

To confirm that the various larger forms of ESET proteins being immunoprecipitated by Oct4 are SUMOylated ESET, we performed immunoprecipitation of ESET from ES cell lysate in the presence of NEM. We found that ESET was indeed SUMOylated as shown by immunoblotting with anti-ESET and anti-SUMO-1 antibodies (Figure 4e, lane 4). Similarly, the immunoprecipitant from anti-SUMO-1 antibody (Figure 4e, lane 5) produced the same, high molecular weight bands as observed with the immunoprecipitant from either anti-ESET (Figure 4e, lane 4) or anti-Oct4 (Figure 4e, lane 3) antibodies. More importantly, these bands were less prominent when immunoprecipitation was carried out without the addition of NEM (Figure 4e, lanes 6 to 9). In addition, the higher molecular weight ESET proteins corresponding to SUMOylated ESET were also observed with the immunoprecipitant from anti-PML antibody (Additional files 4 and 5). Collectively, these data confirmed that the higher molecular weight ESET proteins that interact with Oct4 are SUMOylated ESET suggesting that SUMOylated ESET-Oct4 interaction is required for the repression of *Cdx2*.

Consistent with this, we show by ChIP experiments that SUMO-1 is highly enriched on *Cdx2 *promoter (Figure 4f), most notably at the region that was amplified by primer C7, which happens to be the region where ESET was also most enriched (Figure 2e). The presence of SUMO-1 and ESET at the same region on *Cdx2 *promoter suggests that SUMOylated ESET binds to *Cdx2*, although we cannot rule out a possibility that other SUMOylated proteins might be present in the same region.

### Delocalisation of ESET from PML nuclear bodies enhances the interaction of SUMOylated ESET and Oct4

**Figure 5:**
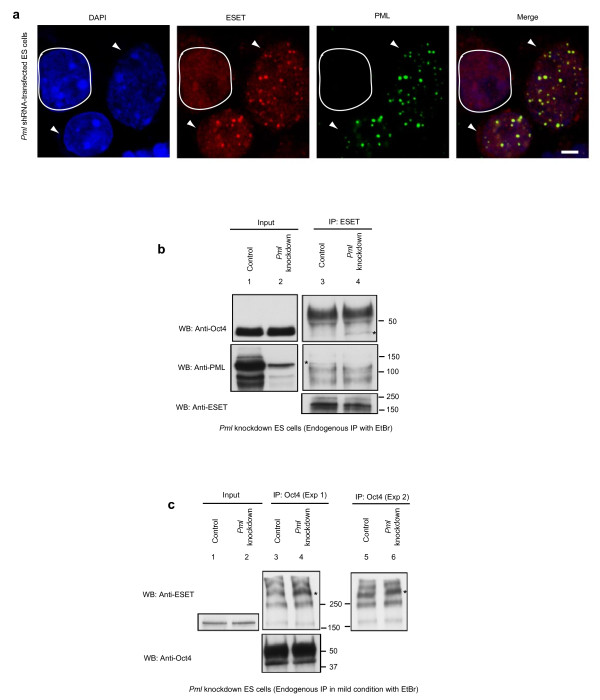
**Delocalisation of ERG-associated protein with SET domain (ESET) from promyelocytic leukaemia (PML) nuclear bodies promotes the interaction of small ubiquitin-related modifier (SUMO)ylated ESET and Oct4**. **(a) **In PML (green)-depleted embryonic stem (ES) cells (white border), ESET (red) punctate foci were delocalised from PML nuclear bodies. Nuclei are labelled in blue. Scale bar, 3 μm. Cultures also contain cells which were not depleted of PML, where ESET exhibit punctate foci (arrow heads) associated with PML bodies. **(b) **ES cells transfected with *Pml *short hairpin RNA (shRNA) for 4 days were immunoprecipitated (IP) with anti-ESET antibody (kind gift of HH Ng; see text) under mild conditions in buffer containing digitonin and ethidium bromide, and were subjected to western blotting (WB) with the antibodies indicated. Note that more Oct4 is precipitated by ESET in *Pml *knockdown ES cells (*lane 4, top panel). Asterisk (lane 3, middle panel) indicates PML being precipitated by ESET in control cells transfected with an empty vector but not in *Pml *knockdown ES cells (compare with lane 4, middle panel). **(c) **ES cells transfected with *Pml *shRNA for 4 days were immunoprecipitated (IP) with anti-Oct4 antibody in buffer containing NP40, *N*-ethylmaleimide, and ethidium bromide and subjected to WB using 4% to 15% Tris-HCl gradient gel. More SUMOylated ESET was precipitated by Oct4 in two independent experiments (Exp 1 and Exp 2) in *Pml *knockdown cells as indicated (*; lane 4 and 6, top panel).

We then considered whether the localisation of ESET to the PML bodies is crucial for its interaction with Oct4. To do this, we performed shRNA knockdown of *Pml *in ES cells. Notably, we found that ESET punctate foci were lost in PML-depleted ES cell nuclei (Figure 5a, white border), although the ESET protein levels remained unaltered (Figure 5c: compare lanes 1 and 2). Furthermore, confocal images suggested that the ESET protein was now uniformly dispersed throughout the ES nuclei. Notably, this delocalisation of ESET from PML nuclear bodies increased its interaction with Oct4 (Figure 5b, lane 4). In the reciprocal immunoprecipitation experiments, more SUMOylated ESET was found to associate with Oct4 (Figure 5c, lanes 4 and 6). This observation suggests that the scaffold composed of PML regulates the interaction between SUMOylated ESET and Oct4, perhaps by modulating the levels of SUMOylated ESET. These results are consistent with our observations that Oct4 preferentially interacts with SUMOylated ESET.

### SUMO-interacting motif (SIM) of Oct4 is crucial for the interaction with ESET and the repression of *Cdx2*

**Figure 6:**
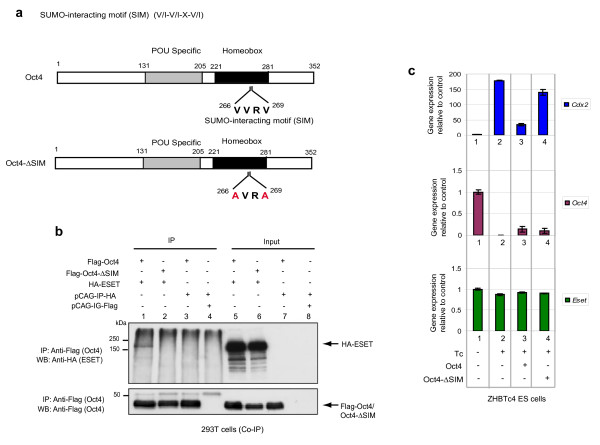
**Small ubiquitin-related modifier (SUMO)-interacting motif (SIM) of Oct4 is crucial for the interaction with ERG-associated protein with SET domain (ESET) and the repression of *Cdx2***. **(a) **Figure depicts SIM in Oct4 (top panel) and the mutations (red alphabet) in the Oct4-lacking SIM (-ΔSIM) (bottom panel). **(b) **Expression vectors indicated were transfected in 293T cells and immunoprecipitant (IP) from anti-Flag antibody and input were subjected to western blot (WB) with anti-haemagglutinin (HA) (ESET, top panel) and anti-Flag (Oct4 or Oct4-ΔSIM, bottom panel) antibodies. **(c) **Quantitative polymerase chain reaction (Q-PCR) analysis of *Cdx2 *(top), *Oct4 *(middle) and *Eset *(bottom) in ZHBTc4 ES cells that were either non-treated or treated with tetracycline (Tc) to deplete the endogenous Oct4 and transfected with the indicated plasmids, relative to control embryonic stem (ES) cells which was set as 1.0 (lane 1) after normalising against *Gapdh*. An empty vector was used as a transfection control in lane 2. Error bars represent standard deviation (SD) of the average and median of four different fractions of cells of different green fluorescent protein (GFP) intensity except for control ES cells (lane 1) where error bar represents the SD of three technical replicates.

To further confirm the interaction of Oct4 and SUMOylated ESET, we attempted to map the sumoylation sites of ESET and the SIM in Oct4. The consensus sumoylation site sequence is ¬-Lys-X-Glu/Asp (¬KxE/D), where ¬ is a large hydrophobic amino acid and X is any amino acid [26]. Based on the sumoylation prediction software, SUMOplot (Abgent, San Diego, California, USA), many putative sumoylation sites were found in the ESET protein (Additional file 6). This is consistent with our observation that SUMOylated ESET exhibited multiple bands when immunoprecipitated with Oct4. Furthermore, this would also explain why both the Tudor domain-containing and SET domain-containing fragments of ESET were found to interact with Oct4 (Additional file 3). Nevertheless, the presence of a large number of putative sumoylation sites in ESET complicates their functional analysis through mutation of these sites. However, only one putative SIM was found in Oct4 (Figure 6a). We thus focussed our studies on investigating the Oct4 SIM.

Proteins that contain the SIM with the consensus Val/Ile-X-Val/Ile-Val/Ile (V/I-X-V/I-V/I) in which X is any amino acid, have a high propensity to interact non-covalently with SUMOylated proteins [27-30]. An inverse SIM (amino acids 266-VVRV-269) was found in Oct4 (Figure 6a). Importantly, when this SIM was mutated in Oct4 (Oct4-ΔSIM), the interaction with ESET was abrogated (Figure 6b, lane 2). This result shows that the SIM of Oct4 is crucial for SUMOylated ESET-Oct4 interaction. The precise nature of this interaction remains to be elucidated.

We then considered if the SIM of Oct4 is functional in the repression of *Cdx2*. We investigated this possibility by substituting the endogenous Oct4 in ZHBTc4 ES cells with exogenous Oct4-ΔSIM. Since the SIM of Oct4 is critical for the interaction with ESET, we anticipated that *Cdx2 *repression would be less effective in cells that express Oct4-ΔSIM compared to cells that express wild-type Oct4. Indeed, this was the case. In control ZHBTc4 ES cells treated with tetracycline, *Cdx2 *was upregulated concomitant with the depletion of Oct4 (Figure 6c, lane 2). When these cells were supplied with exogenous Oct4 (Figure 6c, lane 3), the upregulation of *Cdx2 *was also impeded even though the level of transiently expressed exogenous Oct4 was on average about 10% of the endogenous level in untreated ES cells (Figure 6c, middle panel). However, when Oct4-ΔSIM was supplied instead of wild-type Oct4 (Figure 6c, lane 4) at a comparable level, *Cdx2 *expression was elevated, notably to a level that approaches the *Cdx2 *expression levels induced by the lack of Oct4 (Figure 6c, lane 2). This suggests that the difference in the outcome of response to Oct4 or Oct4-ΔSIM on the repression of *Cdx2 *in the ES cells, might be due to the nature of the repressor complexes that it recruits through its SIM, as Oct4-ΔSIM is able to bind DNA (Additional file 7). It is possible that Oct4 may interact with SUMOylated ESET through the subunits of the transcriptional repression complexes such as HDAC2 [31,32]. We note that *Gata6*, a primitive endoderm-specific gene, as well as *Hand1 *and *Dlx3 *that were upregulated in *Eset *knockdown ES cells (Figure 1d), were similarly repressed by Oct4 and resistant to repression by Oct4-ΔSIM (Additional file 7). These data suggest that SUMOylated ESET-Oct4 complex may act to repress genes other than *Cdx2*. It is equally likely that ESET might interact with other transcription factors to repress a different subset of genes in ES cells. Further work is also required to delineate how Oct4 functions in ES cells to discriminate between its role both as an activator, and as a repressor of genes through ESET-Oct4 complex.

## Conclusion

**Figure 7:**
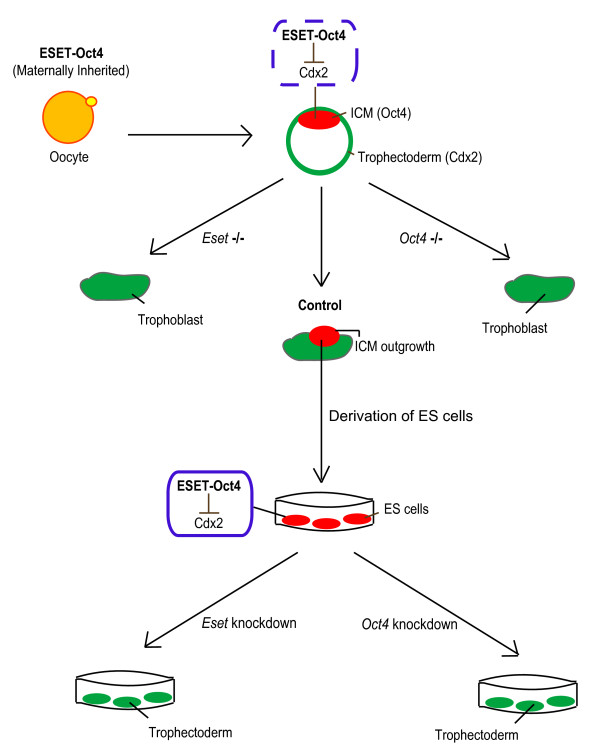
**The proposed model of the *establishment *of pluripotent cells in the inner cell mass (ICM) through the repression of *Cdx2 *by ERG-associated protein with SET domain (ESET)-Oct4 complex**.

We have uncovered an important epigenetic mechanism that maintains pluripotency by preventing differentiation of ES cells, notably into trophectoderm cells. The Oct4-ESET mediated H3K9me3 epigenetic modification involved in the repression of *Cdx2 *may affect other genes, including *Gata6*, to underpin pluripotency. The synergistic action of Oct4 and ESET could also explain the previously described observation that Oct4 regulates expression of *Cdx2 *[12]. The SIM of Oct4 is apparently critical for the interaction of ESET and Oct4, which highlights the importance of this motif in forming an effective SUMOylated ESET-Oct4 repressive complex. Since ESET, like Oct4 is a maternally inherited protein in the oocyte [8], they may also be critical for the *establishment *of pluripotent cells in the ICM, at least in part through repression of *Cdx2 *(Figure 7). Notably, the loss of ESET or Oct4 results in the loss of the pluripotent ICM [8,9]. Furthermore, disruption of the SUMO pathway by the inactivation of the SUMO E2-conjugating enzyme *Ubc9 *is detrimental to the development of the ICM [33]. The use of a conditional allele of *Eset *will address the precise developmental role of this gene in the future. SUMOylated ESET-Oct4 interaction is probably pivotal for both the establishment of pluripotency in the ICM and its maintenance in ES cells.

## Methods

### RNAi constructs and transfection

shRNA oligonucleotides were cloned into the *Bgl*II and *Hin*dIII sites of the pSuper.puro vector (Oligoengine, Seattle, WA, USA). Sequences for *Eset *shRNA which has been described previously [5] are 5'-GATCCCCGATGTGAGTGGATATATCGTTCAAGAGACGATATATCCACTCACATCTTTTTA-3' and 5'-AGCTTAAAAAGATGTGAGTGGATATATCGTCTCTTGAACGATATATCCACTCACATCGGG-3'. Sequences for *Pml *shRNA which has also been described previously [34] are 5'-GATCCCCGCGCAAGTCCAATATCTTCTTCAAGAGAGAAGATATTGGACTTGCGCTTTTTA-3' and 5'-AGCTTAAAAAGCGCAAGTCCAATATCTTCTCTCTTGAAGAAGATATTGGACTTGCGCGGG-3'.

For construction of shRNA-poliovirus internal ribosomal entry site (pIRES)-EGFP, pSuper.puro with or without shRNA insert that has been digested with *Not*I and *Hin*cII were ligated to pIRES-EGFP (Clontech, Mountain View, CA, USA) that has been digested with *Nru*I and *Not*I. For transfection, 0.5 × 10^6 ^ES cells plated in 1 well of a 6-well plate and cultured overnight were transfected with 3 μg of plasmid using Lipofectamine 2000 reagent (Invitrogen, Carlsbad, CA, USA). Transfected cells were selected with 1 μg/ml puromycin (Sigma, St Louis, MO, USA) starting from 24 h after transfection and cells were passaged upon reaching confluency. For alkaline phosphatase staining, western blot and analysis of the gene expression by Q-PCR, cells were harvested on the indicated days without undergoing fluorescence sorting. For morphology visualisation, immunostaining and carrier ChIP analysis, transfected cells were first sorted by FACS either 1 day (for morphology visualisation) or 3 days after transfection. For *Pml *knockdown experiments, immunostaining was performed at day 5 after transfection and immunoprecipitation was performed at day 4 after transfection.

### Cell culture

Undifferentiated ES cells were cultured without feeders on gelatin-coated culture dish in Dulbecco's modified Eagle medium/F12 nutrient mixture without l-glutamine (DMEM/F12) (Gibco, Carlsbad, CA, USA) supplemented with 20% foetal bovine serum (Gibco, Carlsbad, CA, USA), 2 mM l-glutamine (Gibco, Carlsbad, CA, USA), 0.1 mM minimal essential medium (MEM) with non-essential amino acids (Gibco, Carlsbad, CA, USA), 100 U/ml penicillin and 0.1 mg/ml streptomycin (Gibco, Carlsbad, CA, USA), 1 mM sodium pyruvate (Sigma, St Louis, MO, USA), 0.12% sodium bicarbonate solution (Sigma, St Louis, MO, USA), 50 μM 2-mercaptoethanol (Gibco, Carlsbad, CA, USA), 0.15 mM of each nucleoside comprising adenosine, cytidine, guanosine and uridine and 0.05 mM of thymidine (Sigma, St Louis, MO, USA) and 2000 U/ml leukaemia inhibitory factor (Chemicon, Billerica, MA, USA). ZHBTc4 ES cell cultures were as described previously [18]. Oct4 expression was regulated by the addition of 1 μg/ml doxycycline (Sigma, St Louis, MO, USA) to the culture medium. TS cultures were as described previously [13].

### RNA extraction and real-time Q-PCR

Total RNA was prepared using RNeasy Mini Kit (Qiagen, Hilden, Germany) and cDNA was synthesised from 1 μg of RNA using SuperScript III reverse transcriptase (Invitrogen, Carlsbad, CA, USA). Endogenous mRNA levels were measured by Q-PCR based on SYBR Green detection with the ABI Prism 7000 PCR machine (Applied Biosystems, Foster City, CA, USA). Each reaction in a total volume of 20 μl contained 1 μl of 10 × diluted cDNA, 1 μM of forward and reverse primer and 1 × QuantiTect SYBR Green Master Mix reagent (Qiagen, Hilden, Germany). Standard curves for each primer were performed in the same sample plate to determine the relative quantification of the transcript. Q-PCR was performed in triplicates and normalised with *Gapdh*, a housekeeping gene. The data were then normalised against vector control or untreated ES cells which were defined as 100% or 1.0. Each experiment was performed independently on at least two occasions. Sequences of primers used for Q-PCR are available in Additional file 8.

### Protein extraction and immunoblot

Cell lysate was extracted using cold radioimmunoprecipitation assay (RIPA) buffer consisting 50 mM tris(hydroxymethyl)aminomethane (Tris) pH 8.0, 150 mM NaCl, 1% NP40, 0.5% sodium deoxycholate and 0.1% sodium dodecyl sulfate (SDS) added with protease inhibitors (Roche, Basel, Switzerland) for 30 min on ice, followed by centrifugation at 13,000 rpm for 30 min. Supernatant was collected and protein concentration was measured by Bradford assay (Sigma, St Louis, MO, USA). Total protein (20 to 30 μg) was separated by Tris-glycine SDS polyacrylamide gel and transferred to Hybond-P poly(vinylidene difluoride) (PVDF) (Amersham, Buckinghamshire, UK) membrane. Proteins on polyacrylamide gel were visualised by staining with Imperial Protein Stain (Pierce, Waltham, MA, USA).

### Cotransfection and immunoprecipitation

Full details on vector constructions are available in Additional file 9. For cotransfection experiments, 3.5 × 10^6 ^293T cells plated on a 10 cm dish and cultured overnight were transfected with 18 μg of DNA comprising of 9 μg of two different constructs with Lipofectamine 2000 reagent (Invitrogen, Carlsbad, CA, USA). Cells were harvested for immunoprecipitation 48 h after transfection. Cells were washed twice in cold phosphate-buffered saline (PBS) and scraped into 1 ml of PBS. Cell pellet were resuspended in 200 μl of immunoprecipitation buffer (50 mM Tris pH 7.5, 150 mM NaCl, 5 mM ethylenediaminetetraacetic acid (EDTA), 15 mM MgCl2, 0.75% sodium deoxycholate and 1% NP40 added with protease inhibitors from Roche, Basel, Switzerland), incubated on ice for 30 min and centrifuged at 13,000 rpm for 30 min at 4°C. A total of 100 μl of the supernatant was then diluted with 900 μl dilution buffer (50 mM Tris pH 7.5 and 150 mM NaCl added with protease inhibitor from Roche, Basel, Switzerland) so that the final concentration of NP40 in the immunoprecipitation reaction was 0.1%. The primary antibody was incubated with the protein lysate at 4°C overnight. Precipitation was performed by adding 50 μl of Dynabeads Protein G (Invitrogen, Carlsbad, CA, USA) to the reaction for 1 h followed by five washes in buffer containing 50 mM Tris pH 8.0, 150 mM NaCl and 0.1% NP40. Beads were boiled for 5 min with 50 μl 2 × Laemmli sample buffer (Bio-Rad, Hercules, CA, USA) and 20 μl of the supernatant were subjected to western blot. Where indicated, lysis buffer was also added with 20 mM NEM (Sigma, St Louis, MO, USA) and 1:100 diluted phosphatase inhibitors I and II (Sigma, St Louis, MO, USA).

### Endogenous immunoprecipitation

To analyse SUMOylated ESET, one confluent 10 cm dish of ES cells was lysed in 100 μl buffer containing 50 mM Tris pH 7.5, 150 mM NaCl, 5 mM EDTA, 15 mM MgCl2, 0.75% sodium deoxycholate, 1% NP40 supplemented with protease inhibitors (Roche, Basel, Switzerland), 1:100 diluted phosphatase inhibitors I and II (Sigma, St Louis, MO, USA) and 20 mM NEM (Sigma, St Louis, MO, USA). Where indicated, lysis buffer was also added with 50 μg/ml of ethidium bromide. Cell lysate was incubated on ice for 30 min and centrifuged at 13,000 rpm for 30 min at 4°C. A total of 100 μl of the supernatant were then diluted with 900 μl dilution buffer (50 mM Tris pH 7.5 and 150 mM NaCl added with the protease inhibitors (Roche, Basel, Switzerland), 1:100 diluted phosphatase inhibitors I and II (Sigma, St Louis, MO, USA) and 20 mM NEM (Sigma, St Louis, MO, USA). Cell lysate was precleared with 25 μl Dynabeads Protein G (Invitrogen, Carlsbad, CA, USA) for 1 h at 4°C and centrifuged at 13,000 rpm for 20 min at 4°C. The primary antibody was incubated with the supernatant at 4°C overnight. Precipitation was performed by the addition of 50 μl of Dynabeads Protein G to the reaction for 1 h followed by five washes in immunoprecipitation buffer as described above. Beads were resuspended in 35 μl of immunoprecipitation buffer and 45 μl of 2 × Laemmli sample buffer (Bio-Rad, Hercules, CA, USA). After boiling for 5 min, 20 μl of the supernatant were subjected to western blot using 4% to 15% Tris-HCl gradient gel (Bio-Rad, Hercules, CA, USA). For immunoprecipitation in mild conditions (where indicated), lysis and immunoprecipitation buffer consist of 50 mM Tris-HCl pH 7.5, 150 mM NaCl and 0.3% digitonin (Sigma, St Louis, MO, USA) supplemented with protease inhibitors (Roche, Basel, Switzerland), 1:100 diluted phosphatase inhibitors I and II (Sigma, St Louis, MO, USA) and 20 mM NEM (Sigma, St Louis, MO, USA). Wash buffer consists of the same components as immunoprecipitation buffer except that 50 mM NaCl was used.

### Chromatin immunoprecipitation

Chromatin immunoprecipitation was performed according to a published protocol [35] with some modifications. Briefly, cells were crosslinked with 1/10 volume of fresh 11% formaldehyde solution for 15 min and quenched with 1/20 volume of 2.5 M glycine. Cells were sonicated to an average of 500 base pairs (bp) and immunoprecipitated overnight with antibody that was preincubated with 100 μl Dynabeads M-280 Sheep Anti-Rabbit (Invitrogen, Carlsbad, CA, USA) overnight. For isolation of DNA, 100 μl of 10% Chelex (w/v) was added to the washed beads, vortexed and boiled for 10 min [36]. After cooling to room temperature, 100 μg/ml of proteinase K was added and beads were incubated for 30 min at 55°C in a shaking block. Beads were boiled for another 10 min, centrifuged, and the supernatant was collected. The Chelex/bead fraction was vortexed with another 100 μl of water, centrifuged and the supernatant collected was combined with the first supernatant. Immunoprecipitated DNA (3 μl) was used as template for PCR amplification with Red Taq (Sigma, St Louis, MO, USA) or 1 to 2 μl was used for Q-PCR analysis using 1 × QuantiTect SYBR Green Master Mix reagent (Qiagen, Hilden, Germany). Data were normalised to the input or H3 ChIP, control IgG and the least enriched region (as indicated in the figure legends). To compare the enrichment of H3K9me3 between samples, carrier ChIP was performed by adding 3 × 10^7 ^293T cell to 1 × 10^6 ^FACS-sorted, EGFP-positive ES cells transfected with either *Eset *shRNA or empty vector for 3 days (selected with puromycin for 2 days). Carrier ChIP of H3K9me3 was also performed in Figure 3d. Immunoprecipitated DNA was concentrated by ethanol precipitation before PCR analysis. Data were normalised against the input and relative to the controls. Semi quantification of band intensity was performed using Quantity One software (Bio-Rad, Hercules, CA, USA). ChIP experiments were performed independently on at least two occasions. Primers and PCR conditions for ChIP are available in Additional file 10.

### Mutagenesis and transfection

Oct4-ΔSIM construct was generated using the QuikChange XL Site-Directed Mutagenesis Kit (Stratagene, La Jolla, CA, USA). Details on the construct are available in Additional file 9. For transfection, 0.5 × 10^6 ^ZHBTc4 ES cells plated on 1 well of a 6-well plate and cultured overnight were transfected with 3 μg of plasmid using Lipofectamine 2000 reagent (Invitrogen, Carlsbad, CA, USA). Doxycycline 1 μg/ml (Sigma, St Louis, MO, USA) was added to the culture medium after 6 h of transfection to remove the endogenous Oct4. Transfected cells were sorted by FACS after 48 h of transfection.

### Immunofluorescence microscopy

Single cells on poly-l-lysine (Sigma, St Louis, MO, USA) treated 12 well microscope slides (Erie Scientific Company, Waltham, MA, USA) were centrifuged at 1,000 rpm for 1 min and fixed with 4% paraformaldehyde (Sigma, St Louis, MO, USA) in PBS for 15 min. This was followed by three washes in PBS and blocking and permeabilisation in 0.1% Triton X (v/v) and 1% bovine serum albumin (BSA) (w/v) in PBS for 30 min. Primary antibodies diluted in blocking buffer were incubated at 4°C overnight. Secondary antibodies conjugated to Alexa Fluor 488 and 568 (Molecular Probes, Carlsbad, CA, USA) and DAPI (1 μg/ml) were incubated at room temperature, in the dark for 1 h. Cells were mounted on slides with Vectashield (Vector Laboratories, Burlingame, CA, USA) containing DAPI and images were captured with a Bio-Rad Radiance 2000 confocal microscope.

### Antibodies

Antibodies used for western blot were; ESET (07-378, Upstate, Billerica, MA, USA), α-tubulin (T9026, Sigma, St Louis, MO, USA), H3K9me3 (07-442, Upstate, Billerica, MA, USA), H3K4me2 (ab7766, Abcam, Cambridge, UK), Oct-3 (611202, BD, Franklin Lakes, NJ, USA), Cdx2 (Biogenex, San Ramon, CA, USA), SUMO-1 (Affinity Bioreagents, Waltham, MA, USA), PML (05-718, Upstate, Billerica, MA, USA) Anti-Flag (F3165, Sigma, St Louis, MO, USA) and Anti-HA (ab9110; Abcam, Cambridge, UK). Antibodies used for immunoprecipitation were; ESET (ab12317, Abcam, Cambridge, UK), (kind gift from HH Ng, Genome Institute of Singapore, Singapore) and (sc-46110, Santa Cruz, Santa Cruz, CA, USA), SUMO-1 (Affinity Bioreagents, Waltham, MA, USA), PML (sc-5621, Santa Cruz, Santa Cruz, CA, USA), Oct4 (sc-9081, Santa Cruz, Santa Cruz, CA, USA), Anti-Flag (F3165, Sigma, St Louis, MO, USA) and Anti-HA (ab9110; Abcam, Cambridge, UK).

Antibodies used for chromatin immunoprecipitation were; H3K9me3 (ab8898; Abcam, Cambridge, UK), ESET (kind gift from HH Ng, Genome Institute of Singapore), Oct4 (sc-9081, Santa Cruz, Santa Cruz, CA, USA), SUMO-1 (Zymed, Carlsbad, CA, USA), Anti-Flag (F3165, Sigma, St Louis, MO, USA) and Anti-HA (ab9110; Abcam, Cambridge, UK). Antibodies used for immunofluorescence were Cdx2 (1:100, Biogenex, San Ramon, CA, USA), ESET (1:100, 07-378, Upstate, Billerica, MA, USA) and PML (1:200, 05-718, Upstate, Billerica, MA, USA).

## Competing interests

The authors declare that they have no competing interests.

## Authors' contributions

L-SY, KH and MAS were involved in project planning and data analysis. L-SY performed experimental work. L-SY and MAS wrote the manuscript.

## Supplementary Material

Additional file 1 ***Eset *is required for normal embryonic stem (ES) cell phenotype**. **(a) **Alkaline phosphatase staining of *Eset *knockdown (right panel) and control (left panel) ES cells after 7 days of short hairpin RNA (shRNA) transfection. Scale bar, 100 μm. **(b) **Morphology of *Eset *knockdown and control ES cells after 3 days of shRNA transfection from both EGFP-positive (transfected) and EGFP-negative (untransfected) populations. Scale bar, 100 μm. Inset, 50 μm. Click here for file

Additional file 2 **ERG-associated protein with SET domain (ESET)-mediated histone 3 lysine 9 trimethylation (H3K9me3) represses *Cdx2 *in embryonic stem (ES) cells**. **(a) **Semiquantitative polymerase chain reaction (PCR)-chromatin immunoprecipitation (ChIP) analysis of H3K9me3 at the *Cdx2 *and *Oct4 *promoter regions in ES cells. **(b) **Carrier ChIP semiquantitative PCR analysis of H3K9me3 in fluorescence-activated cell sorting (FACS)-sorted *Eset *knockdown ES cells (+) and control ES cells (-). 293T cells were added as carrier. H3K9me3 levels were downregulated at the *Cdx2 *promoter region (top panel, lane 4). No signal was detected in carrier cells, 293T input (lane 7) showing specificity of PCR to mouse genomic DNA. Primers C6 and C7 were not specific to mouse DNA. H3K9me3 was not detected at the control *Oct4 *promoter region (middle panel). Suv39h1/2-mediated H3K9me3 level at the major satellite region was unaffected in ESET-depleted ES cells (bottom panel). Click here for file

Additional file 3 **ERG-associated protein with SET domain (ESET) binding to *Cdx2 *promoter is dependent on Oct4 through Oct4-ESET interaction**. **(a) **Quantitative polymerase chain reaction (Q-PCR) analysis of the levels of haemagglutinin (HA)-ESET enrichment at region C7 of the *Cdx2 *promoter in ZHBTc4 embryonic stem (ES) cells, which were first treated with tetracycline, followed 1 day later by transfection of HA-ESET. At 1 day after transfection (day 2 of tetracycline treatment), cells were harvested for ChIP experiments. HA-ESET enrichment on day 2 of tetracycline treatment is relative to untreated cells after normalising against ZHBTc4 ES cells transfected with an empty vector, and their respective input. Error bars, standard deviation (SD) of three technical replicates. **(b) **Coimmunoprecipitation of ESET with Oct4 in 293T cells. Expression vectors indicated were transfected and Flag-tagged Oct4 protein was immunoprecipitated. Immunoprecipitant (IP) and supernatant were subjected to western blot (WB) with anti-HA (ESET, top panel) and anti-Flag (Oct4, bottom panel) antibodies. HA, haemagglutinin. **(c) **Drawings depicting full length ESET and ESET mutant proteins. Numbers indicate amino acids. **(d) **Coimmunoprecipitation of ESET-ΔSET and ESET-ΔTudor with Oct4 in 293T cells. Expression vectors indicated were transfected and Flag-tagged Oct4 protein was immunoprecipitated. Click here for file

Additional file 4 **Interaction of small ubiquitin-related modifier (SUMO)ylated ERG-associated protein with SET domain (ESET) and Oct4 is DNA independent**. **(a) **Embryonic stem (ES) cell lysates were immunoprecipitated (IP) with anti-ESET antibody (kind gift of HH Ng; see text) under mild conditions in digitonin-containing buffer in the presence of 50 μg/ml ethidium bromide and subjected to western blotting (WB) with the antibodies indicated. Rabbit IgG was used as a negative control. **(b) **ES cell lysates were immunoprecipitated (IP) with the indicated antibodies in buffer containing NP40 and *N*-ethylmaleimide either in the presence or absence of 50 μg/ml ethidium bromide and subjected to WB using 4% to 15% Tris-HCl gradient gel. A rabbit anti-haemagglutinin (HA) antibody was used as a negative control. Click here for file

Additional file 5 **ERG-associated protein with SET domain (ESET) interacts with promyelocytic leukaemia (PML)**. **(a) **Coimmunoprecipitation of PML with ESET in 293T cells. Immunoprecipitant (IP) and supernatant were subjected to western blot (WB) with anti-Flag (PML, top panel) and anti-haemagglutinin (HA) (ESET, bottom panel) antibodies. **(b) **Embryonic stem (ES) cell lysates were immunoprecipitated (IP) with the indicated antibodies in buffer containing NP40 in the presence of *N*-ethylmaleimide (NEM) and subjected to WB using 4% to 15% Tris-HCl gradient gel. A rabbit anti-HA antibody was used as a negative control. Click here for file

Additional file 6 **Putative small ubiquitin-related modifier (SUMO)ylation sites of ERG-associated protein with SET domain (ESET) based on the SUMOplot software**. Red triangles represent motifs with high probability and blue triangles represent motifs with low probability. Click here for file

Additional file 7 **Mutation of Oct4 SIM does not affect its ability to bind DNA**. **(a) **Quantitative polymerase chain reaction (Q-PCR) analysis of the levels of Flag-tagged enrichment at region O1 of the *Oct4 *promoter in ZHBTc4 ES cells transfected with Flag-Oct4 or Flag-Oct4-ΔSIM relative to controls transfected with an empty vector after normalising against their respective input. Tetracycline was added to the culture medium 6 h after transfection to deplete endogenous Oct4. Cells were harvested at 48 h after transfection. Error bars, standard deviation (SD) of three technical replicates. **(b) **Q-PCR analysis of *Gata6 *(top), *Dlx3 *(middle) and *Hand1 *(bottom) in ZHBTc4 embryonic stem (ES) cells; these cells were treated with tetracycline (Tc+) to deplete the endogenous Oct4, or left untreated (Tc-). They were transfected with the indicated control or mutant Oct4 plasmids. Gene expression levels are relative to control ES cells which was set as 1.0 (lane 1) after normalising against *Gapdh*. An empty vector was used as a transfection control in lane 2. Error bars represent SD of the average and median of four different fractions of cells of different GFP intensity except for control ES cells (lane 1) where error bar represents the SD of three technical replicates. Click here for file

Additional file 8 **Table S1**. Quantitative polymerase chain reaction (Q-PCR) Primers Click here for file

Additional file 9 **Supplementary methods**. Overexpression constructs Click here for file

Additional file 10 **Table S2**. Chromatin Immunoprecipitation (ChIP) Primers Click here for file
