# Supplementary figures and images for: ERG-associated protein with SET domain (ESET)-Oct4 interaction regulates pluripotency and represses the trophectoderm lineage
Source: Epigenetics Chromatin. 2009 Oct 7;2:12. doi: 10.1186/1756-8935-2-12 (PMC2763847; doi:10.1186/1756-8935-2-12)

Supplementary Figure 1 - Surani

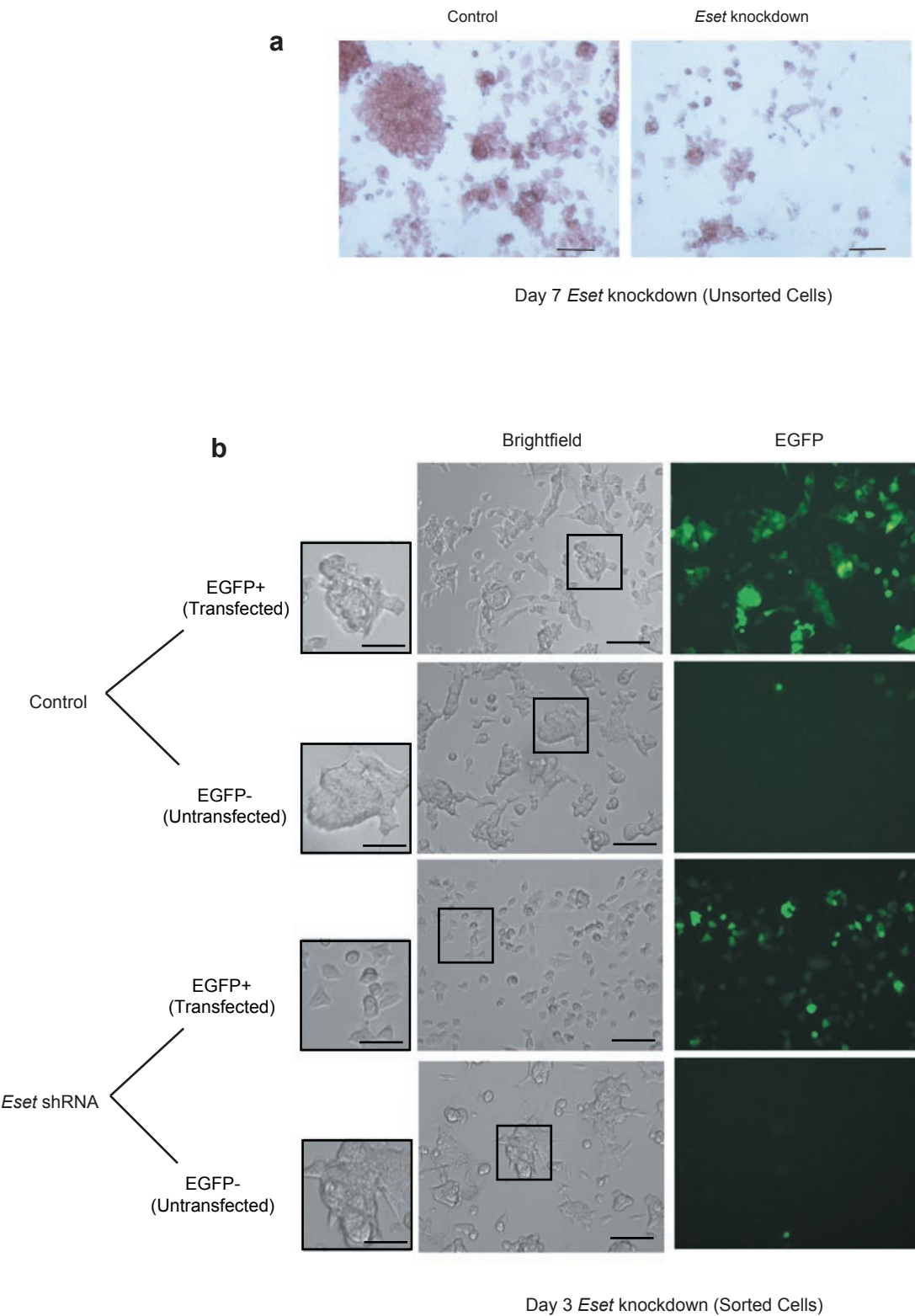

Supplement: Additional file 1 — Eset is required for normal embryonic stem (ES) cell phenotype. (a) Alkaline phosphatase staining of Eset knockdown (right panel) and control (left panel) ES cells after 7 days of short hairpin RNA (shRNA) transfection. Scale bar, 100 μm. (b) Morphology of Eset knockdown and control ES cells after 3 days of shRNA transfection from both EGFP-positive (transfected) and EGFP-negative (untransfected) populations. Scale bar, 100 μm. Inset, 50 μm. [file 1756-8935-2-12-S1.PDF]

Supplementary Figure 2 - Surani

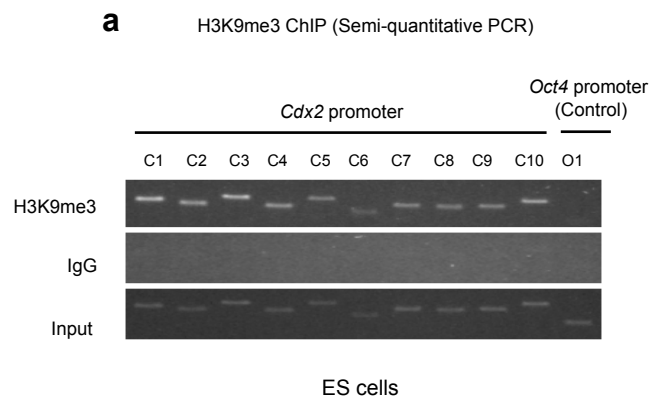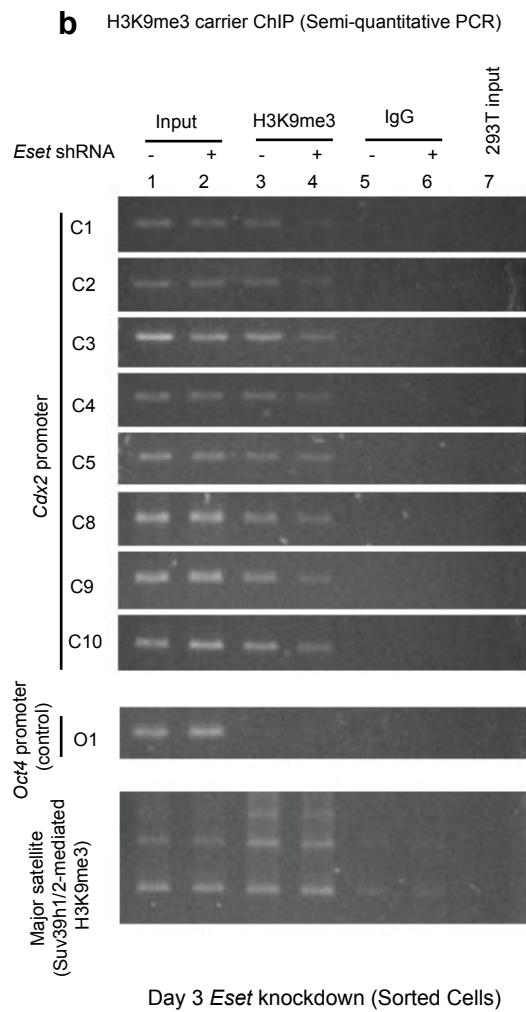

Supplement: Additional file 2 — ERG-associated protein with SET domain (ESET)-mediated histone 3 lysine 9 trimethylation (H3K9me3) represses Cdx2 in embryonic stem (ES) cells. (a) Semiquantitative polymerase chain reaction (PCR)-chromatin immunoprecipitation (ChIP) analysis of H3K9me3 at the Cdx2 and Oct4 promoter regions in ES cells. (b) Carrier ChIP semiquantitative PCR analysis of H3K9me3 in fluorescence-activated cell sorting (FACS)-sorted Eset knockdown ES cells (+) and control ES cells (-). 293T cells were added as carrier. H3K9me3 levels were downregulated at the Cdx2 promoter region (top panel, lane 4). No signal was detected in carrier cells, 293T input (lane 7) showing specificity of PCR to mouse genomic DNA. Primers C6 and C7 were not specific to mouse DNA. H3K9me3 was not detected at the control Oct4 promoter region (middle panel). Suv39h1/2-mediated H3K9me3 level at the major satellite region was unaffected in ESET-depleted ES cells (bottom panel). [file 1756-8935-2-12-S2.PDF]

Supplementary Figure 3 - Surani

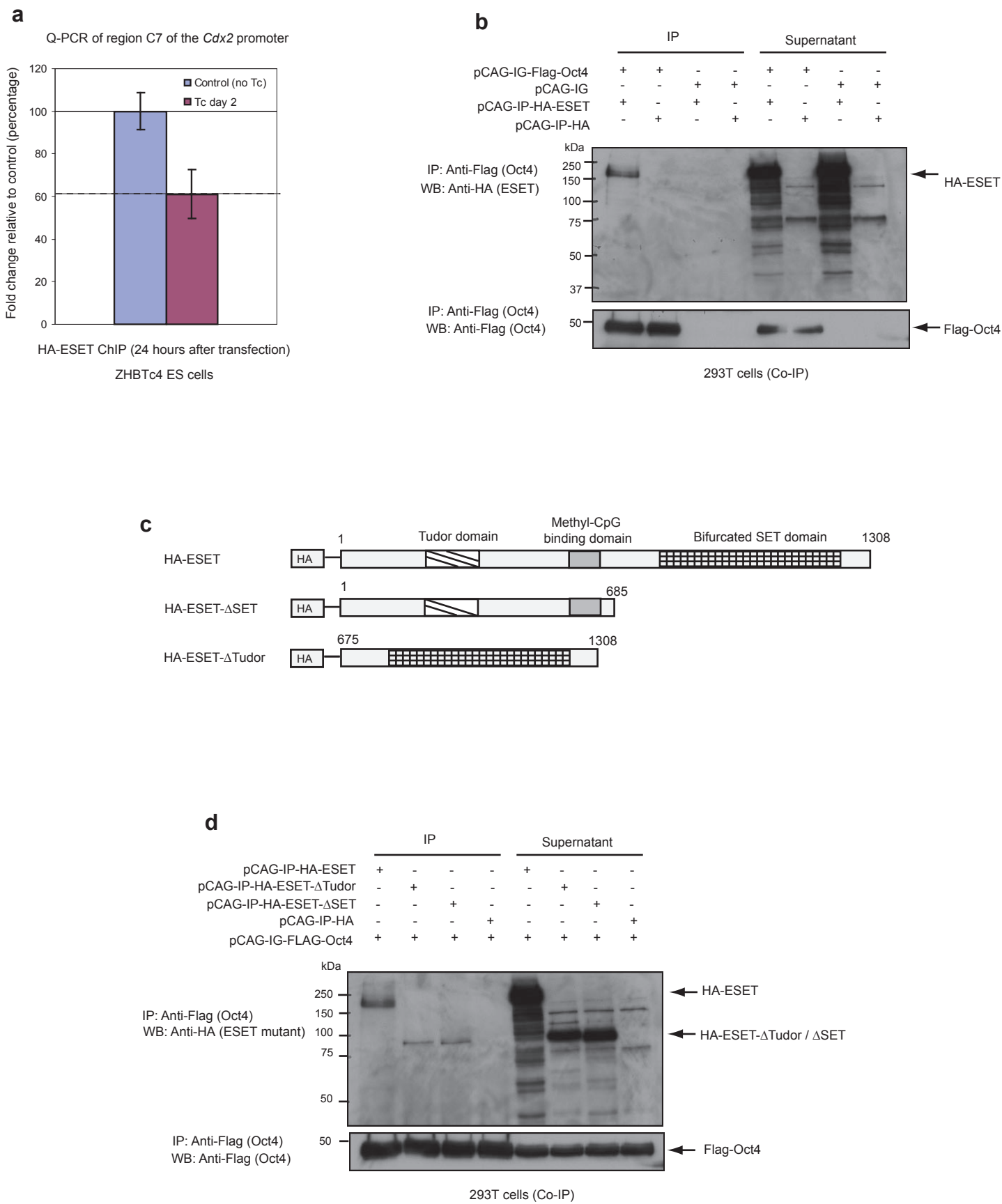

Supplement: Additional file 3 — ERG-associated protein with SET domain (ESET) binding to Cdx2 promoter is dependent on Oct4 through Oct4-ESET interaction. (a) Quantitative polymerase chain reaction (Q-PCR) analysis of the levels of haemagglutinin (HA)-ESET enrichment at region C7 of the Cdx2 promoter in ZHBTc4 embryonic stem (ES) cells, which were first treated with tetracycline, followed 1 day later by transfection of HA-ESET. At 1 day after transfection (day 2 of tetracycline treatment), cells were harvested for ChIP experiments. HA-ESET enrichment on day 2 of tetracycline treatment is relative to untreated cells after normalising against ZHBTc4 ES cells transfected with an empty vector, and their respective input. Error bars, standard deviation (SD) of three technical replicates. (b) Coimmunoprecipitation of ESET with Oct4 in 293T cells. Expression vectors indicated were transfected and Flag-tagged Oct4 protein was immunoprecipitated. Immunoprecipitant (IP) and supernatant were subjected to western blot (WB) with anti-HA (ESET, top panel) and anti-Flag (Oct4, bottom panel) antibodies. HA, haemagglutinin. (c) Drawings depicting full length ESET and ESET mutant proteins. Numbers indicate amino acids. (d) Coimmunoprecipitation of ESET-ΔSET and ESET-ΔTudor with Oct4 in 293T cells. Expression vectors indicated were transfected and Flag-tagged Oct4 protein was immunoprecipitated. [file 1756-8935-2-12-S3.PDF]

Supplementary Figure 4- Surani

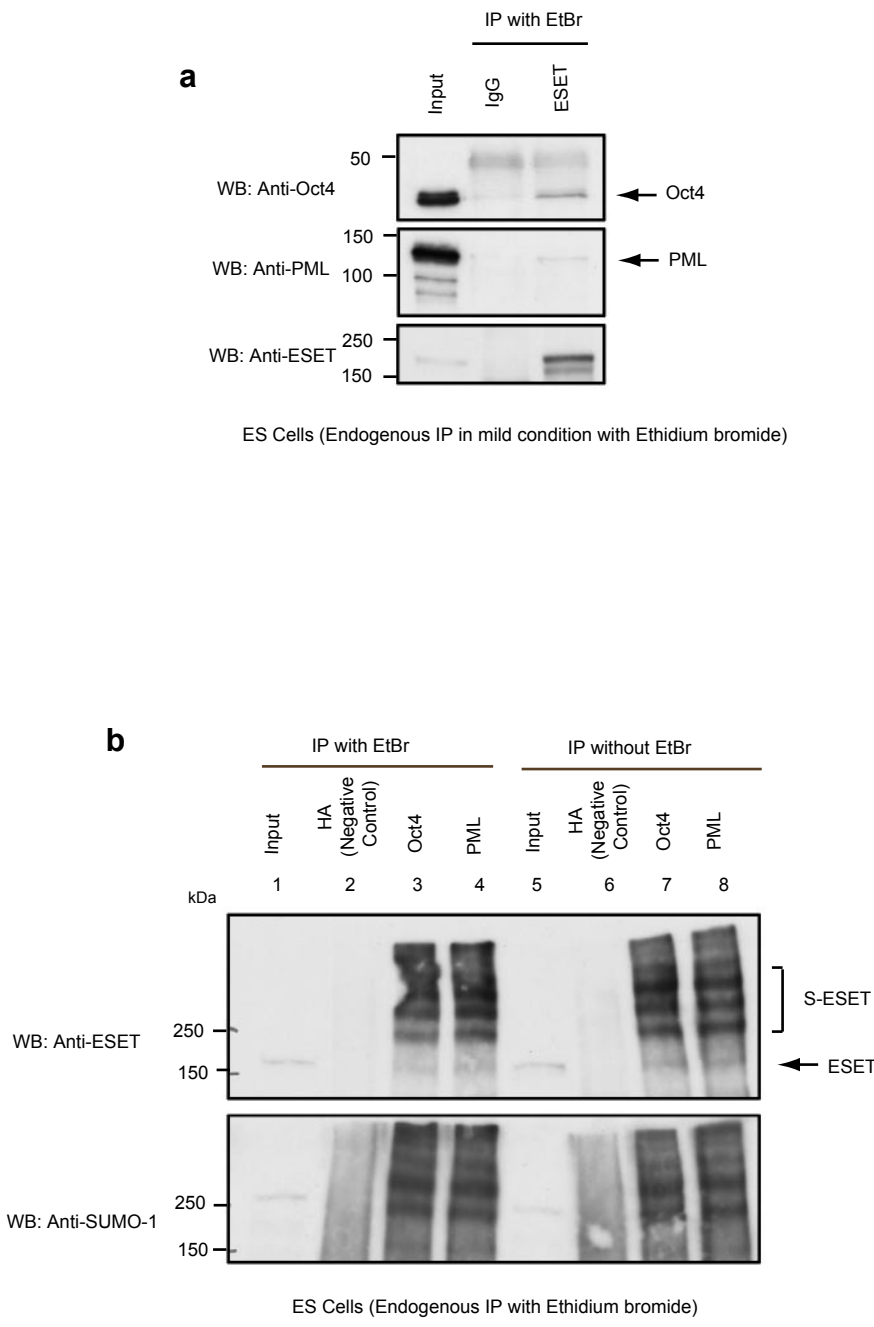

Supplement: Additional file 4 — Interaction of small ubiquitin-related modifier (SUMO)ylated ERG-associated protein with SET domain (ESET) and Oct4 is DNA independent. (a) Embryonic stem (ES) cell lysates were immunoprecipitated (IP) with anti-ESET antibody (kind gift of HH Ng; see text) under mild conditions in digitonin-containing buffer in the presence of 50 μg/ml ethidium bromide and subjected to western blotting (WB) with the antibodies indicated. Rabbit IgG was used as a negative control. (b) ES cell lysates were immunoprecipitated (IP) with the indicated antibodies in buffer containing NP40 and N-ethylmaleimide either in the presence or absence of 50 μg/ml ethidium bromide and subjected to WB using 4% to 15% Tris-HCl gradient gel. A rabbit anti-haemagglutinin (HA) antibody was used as a negative control. [file 1756-8935-2-12-S4.PDF]

Supplementary Figure 5- Surani

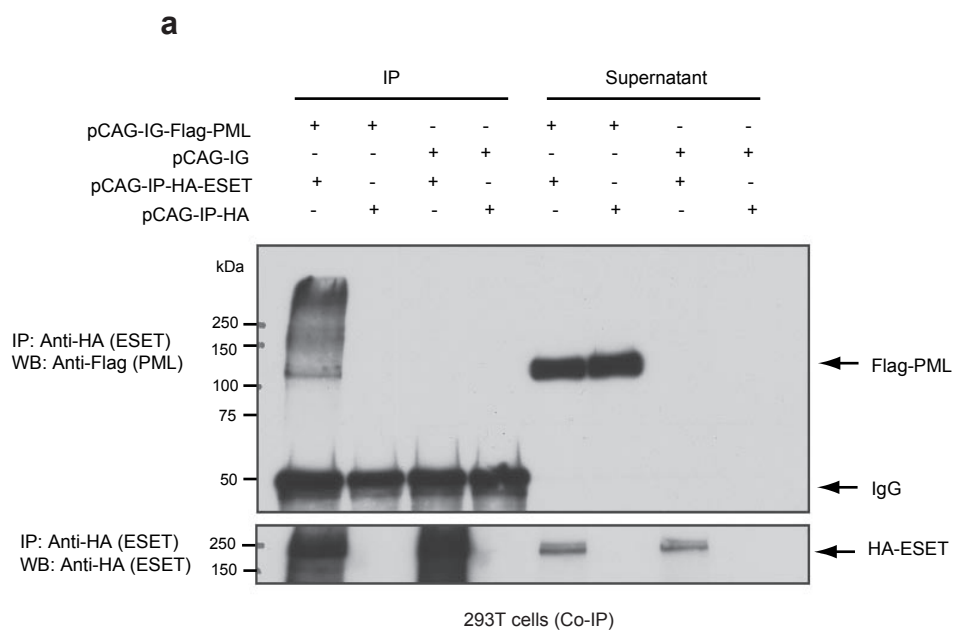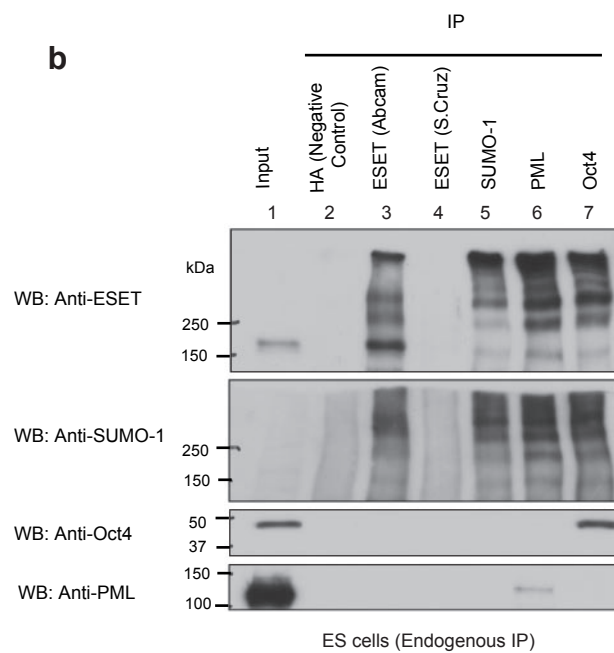

Supplement: Additional file 5 — ERG-associated protein with SET domain (ESET) interacts with promyelocytic leukaemia (PML). (a) Coimmunoprecipitation of PML with ESET in 293T cells. Immunoprecipitant (IP) and supernatant were subjected to western blot (WB) with anti-Flag (PML, top panel) and anti-haemagglutinin (HA) (ESET, bottom panel) antibodies. (b) Embryonic stem (ES) cell lysates were immunoprecipitated (IP) with the indicated antibodies in buffer containing NP40 in the presence of N-ethylmaleimide (NEM) and subjected to WB using 4% to 15% Tris-HCl gradient gel. A rabbit anti-HA antibody was used as a negative control. [file 1756-8935-2-12-S5.PDF]

Supplementary Figure 7- Surani

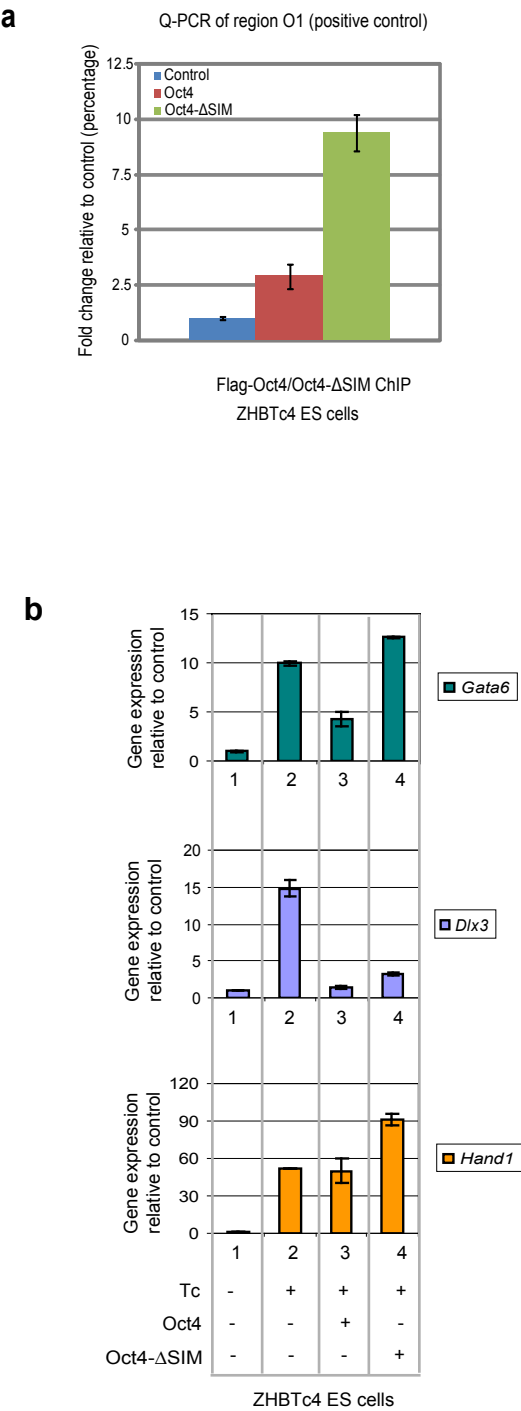

Supplement: Additional file 7 — Mutation of Oct4 SIM does not affect its ability to bind DNA. (a) Quantitative polymerase chain reaction (Q-PCR) analysis of the levels of Flag-tagged enrichment at region O1 of the Oct4 promoter in ZHBTc4 ES cells transfected with Flag-Oct4 or Flag-Oct4-ΔSIM relative to controls transfected with an empty vector after normalising against their respective input. Tetracycline was added to the culture medium 6 h after transfection to deplete endogenous Oct4. Cells were harvested at 48 h after transfection. Error bars, standard deviation (SD) of three technical replicates. (b) Q-PCR analysis of Gata6 (top), Dlx3 (middle) and Hand1 (bottom) in ZHBTc4 embryonic stem (ES) cells; these cells were treated with tetracycline (Tc+) to deplete the endogenous Oct4, or left untreated (Tc-). They were transfected with the indicated control or mutant Oct4 plasmids. Gene expression levels are relative to control ES cells which was set as 1.0 (lane 1) after normalising against Gapdh. An empty vector was used as a transfection control in lane 2. Error bars represent SD of the average and median of four different fractions of cells of different GFP intensity except for control ES cells (lane 1) where error bar represents the SD of three technical replicates. [file 1756-8935-2-12-S7.PDF]
