# Supplementary material for: ERG-associated protein with SET domain (ESET)-Oct4 interaction regulates pluripotency and represses the trophectoderm lineage
Source: Epigenetics Chromatin. 2009 Oct 7;2:12. doi: 10.1186/1756-8935-2-12 (PMC2763847; doi:10.1186/1756-8935-2-12)

Supplementary Figure 6 - Surani

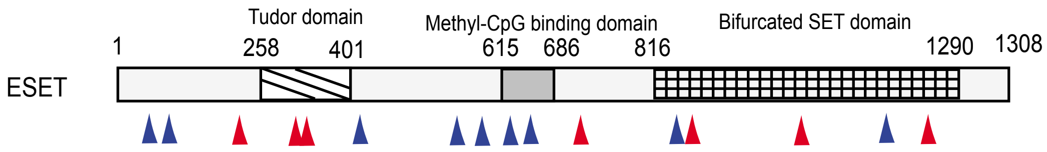

SUMOylation sites (ΨKxE/D)

Ψ - hydrophobic amino acid
x - any amino acid

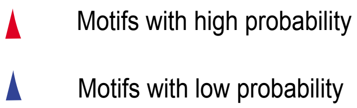

Supplement: Additional file 6 — Putative small ubiquitin-related modifier (SUMO)ylation sites of ERG-associated protein with SET domain (ESET) based on the SUMOplot software. Red triangles represent motifs with high probability and blue triangles represent motifs with low probability. [file 1756-8935-2-12-S6.PDF]
