# Supplementary material for: ERG-associated protein with SET domain (ESET)-Oct4 interaction regulates pluripotency and represses the trophectoderm lineage
Source: Epigenetics Chromatin. 2009 Oct 7;2:12. doi: 10.1186/1756-8935-2-12 (PMC2763847; doi:10.1186/1756-8935-2-12)
Supplement: Additional file 8 — Table S1. Quantitative polymerase chain reaction (Q-PCR) Primers [file 1756-8935-2-12-S8.DOC]

**SUPPLEMENTARY TABLE 1**

| **Primer Name** | **Sequences** | **Cycles** |
| --- | --- | --- |
| **Q-PCR Primers** | | |
| Eset | CCATTGAATGCAGAGGGAGACT TGGGGTCAGAGTTCAACAGTCA | 40 |
| Oct4 | AGATCACTCACATCGCCAATCA CGCCGGTTACAGAACCATACTC | 40 |
| Nanog | AGGCTTTGGAGACAGTGAGGTG TGGGTAAGGGTGTTCAAGCACT | 40 |
| Sox2 | AAACTTTTGTCCGAGACCGAGA TTATAATCCGGGTGCTCCTTCA | 40 |
| Cdx2 | TTATGGACCTCAGGGGAAGACA GAAGAAGCCCCAGGAATCACTT | 40 |
| Hand1 | TTCCCCTCTTCCGTCCTCTTAC AAATTCAGCAACGAATGGGAAC | 40 |
| Dlx3 | TTGCAAGTCGAAAGAGGGATGT CCCACCACGTCTGTCTCTTCTT | 40 |
| Ets2 | TTACCAGCATGGTGACCTCAGA GTCCCGTGTGGTCTCATGGTAT | 40 |
| Eomes | GATGGACCTGGTGGTGTTTTGT CATCAAAGGTGGAAGGCAAAAG | 40 |
| Gata4 | TTCCTCTCCCAGGAACATCAAA GCTGCACAACTGGGCTCTACTT | 40 |
| Gata6 | TGCAAGATTGCATCATGACAGA TGACCTCAGATCAGCCACGTTA | 40 |
| Fgf5 | AAACTCCATGCAAGTGCCAAAT TCTCGGCCTGTCTTTTCAGTTC | 40 |
| T | TGCACATTACACACCACTGACG AGAACCAGAAGACGAGGACGTG | 40 |
| Gapdh | GCGGCACGTCAGATCCA CATGGCCTTCCGTGTTCCT | 40 |
