# Supplementary material for: ERG-associated protein with SET domain (ESET)-Oct4 interaction regulates pluripotency and represses the trophectoderm lineage
Source: Epigenetics Chromatin. 2009 Oct 7;2:12. doi: 10.1186/1756-8935-2-12 (PMC2763847; doi:10.1186/1756-8935-2-12)
Supplement: Additional file 9 — Supplementary methods. Overexpression constructs [file 1756-8935-2-12-S9.DOC]

**METHODS**

**Over-expression Constructs**

pCAG-IP-HA was constructed by annealing oligonucleotides containing 3 x HA with SalI and NotI restriction sites at both ends into pCAG-IP (kind gift from Austin Smith) that was digested with XhoI and NotI. A XhoI restriction site is included downstream of the tag such that inserts can be clone into the XhoI and NotI sites. The sequences of the tag are:

5’TCGACATGTACCCATACGACGTCCCAGACTACGCTTACCCATACGACGTCCCAGACTACG CTTACCCATACGACGTCCCAGACTACGCTCTCGAGACCTTGGC-3’ and

5’GGCCGCCAAGGTCTCGAGAGCGTAGTCTGGGACGTCGTATGGGTAAGCGTAGTCTGGGA CGTCGTATGGGTAAGCGTAGTCTGGGACGTCGTATGGGTACATG-3’

Full-length *Eset* without stop codon was cloned from mouse embryonic stem cells cDNA

using Pfx DNA polymerase (Invitrogen) and the following primers:

5’- ATTGTCGACATGTCCTCCCTCCCTGGGTGCATG- 3’ and

5’-AGAATCGATAAGAAGTCTCCCTCTGCATTCAAT- 3’

The resulting PCR product was cloned into Zero Blunt® TOPO® PCR Cloning Kit (Invitrogen) and sequence were verified. To generate pCAG-IP-HA-ESET, a linker with a stop codon, Linker-ESET with EcoRI and NotI sticky ends was ligated with pCAG-IP- HA digested with XhoI and NotI and TOPO-ESET digested with SalI and EcoRI. For pCAG-IP-HA-control, a linker with stop codon, Linker-control with SalI and NotI sticky ends was ligated with pCAG-IP-HA digested with XhoI and NotI.

Sequences of Linker-ESET are;

5’-AATTCTAACCGCGGGAGCTCGC-3’ and 5’- GGCCGCGAGCTCCCGCGGTTAG-3’. Sequences of Linker-control are;

5’- TCGACTAACCGCGGGAGCTCGC-3’ and 5’-GGCCGCGAGCTCCCGCGGTTAG-3’.

To generate HA-ESET-∆Tudor, pCAG-IP-HA digested with XhoI and NotI was ligated with pCAG-IP-HA-ESET digested with NdeI and NotI and a linker that contains XhoI and NdeI sticky ends; Linker-∆Tudor.

Sequences of Linker-∆Tudor are;

5’-TCGAGATGTTCTGTTTGGATCCA-3’ and 5’-TATGGATCCAAACAGAACATC-3’.

To generate HA-ESET-∆SET, pCAG-IP-HA digested with XhoI and NotI was ligated with TOPO-ESET digested with SalI and NdeI and a linker that contains a stop codon; Linker-∆SET with NdeI and NotI sticky ends.

Sequences of Linker-∆Set are;

5’-TATGTTCTTGTTGACTAAGC-3’ and 5’-GGCCGCTTAGTCAACAAGAACA-3’.

To generate pCAG-IG-Flag-Oct4, the following primers were used to amplify full length *Oct4* from mouse embryonic stem cells cDNA and cloned into pCAG-IG at XhoI and NotI sites;

5’ATTGTCGACCACCATGGATTACAAGGATGACGACGATAAGATGGCTGGACACCTGGCTTC AGACTTCG-3’ and 5’-GTAGCGGCCGCTTAACCCCAAAGCTCCAGGTTCTCT-3’.

To generate pCAG-IG-Flag-Oct4-∆SIM, the following oligonucleotide primers were used to synthesis the mutant strand with pCAG-IG-Flag-Oct4 as the template.

5’ GGCTAGAGAAGGATGCGGCTCGAGTATGGTTCTG-3’ and

5’ CAGAACCATACTCGAGCCGCATCCTTCTCTAGCC-3’

To generate pCAG-IG-Flag-PML, the following primers were used to amplify full length *Pml* from mouse embryonic stem cells cDNA, and the 2.5 kb product was cloned into pCAG-IG at XhoI and NotI sites;

5’ATTGTCGACCACCATGGATTACAAGGATGACGACGATAAGATGCCTCCCCCAGAGGAACC CTCCGAAG-3 and’ 5’-GTAGCGGCCGCCTAGGCCAGGCATCCCTTACTTTCA-3’

To generate pCAG-IG-Flag-SUMO-1, the following primers were used to amplify

SUMO-1 from human brain cDNA and cloned into pCAG-IG at XhoI and NotI sites;

5’ATTGTCGACCACCATGGATTACAAGGATGACGACGATAAGATGTCTGACCAGGAGGCAAA ACCTTCAA-3’ and 5’-GTAGCGGCCGCCTAACCCCCCGTTTGTTCCTGATAAACTTCA-3’
