# Supplementary material for: ERG-associated protein with SET domain (ESET)-Oct4 interaction regulates pluripotency and represses the trophectoderm lineage
Source: Epigenetics Chromatin. 2009 Oct 7;2:12. doi: 10.1186/1756-8935-2-12 (PMC2763847; doi:10.1186/1756-8935-2-12)
Supplement: Additional file 10 — Table S2. Chromatin Immunoprecipitation (ChIP) Primers [file 1756-8935-2-12-S10.DOC]

**SUPPLEMENTARY TABLE 2**

| **Primer Name** | **Sequences** | **Cycles** |
| --- | --- | --- |
| **ChIP Primers** | | |
| C1 | TGAGCAGCTAAAGGCCCTTCTTACTCCT AATCTGTCCTCAGCCACTCTACTCCATCG | 27a  32b  40c |
| C2 | AGGTCCTGTCACAGTAGCTGGAACAGACA TTTGAAGAGGGTCTCTTTTCAGCATCCAG | 27a  32b  40c |
| C3 | GAAGATCGAGTCAGAGACCACCACTCCTT TTGGGAGACCGACAGAGCTACTGTACTCA | 27a  32b  40c |
| C4 | GGAAAGTAAGATGACACCCTTGCTGTTCC CCTCAATTCCCACCTCTTGCATTTCTTAC | 27a  32b |
| C5 | TTCCGTAGCAGTAATGAATAGCGACAACG CCAGAACTCCAGCCCAAGTAGATATGACA | 27a  32b  40c |
| C6 | ATAAATGTATTGCTGAGGGTTGTCCGACC CTTTCGTACATTCTCAGGCAGAAGGTGGT | 27a |
| C7 | CTGTGATTGGAGGTTAAAGTGCACCCAG AGTAATGACACAGACACCAATGGCTGGAG | 27a  40c |
| C8 | GTAAACACTCGTTAATCACGTAAGGCCGC AGAGATAAAGGTAGCTGGCTGCACCTCAG | 27a  32b  40c |
| C9 | CTGAGGTGCAGCCAGCTACCTTTATCTCT GTACTGCGGAGGACTGACAAAGTTCTGC | 27a  32b  40c |
| C10 | GAAATACAGGTGGATTCATCCCAGGAGTG AGGTAGCTAAGTTGAGTAGGCACGGCAAA | 27a  32b  40c |
| O1[1] | GGAACTGGGTGTGGGGAGGTTGTA AGCAGATTAAGGAAGGGCTAGGACGAGAG | 27a  32b  40c |
| Major satellite  [2] | GACGACTTGAAAAATGACGAAATC CATATTCCAGGTCCTTCAGTGTGC | 21b |

a ChIP-semi quantitative PCR

b Carrier ChIP –semi quantitative PCR

c ChIP/Carrier ChIP–Q-PCR

**SUPPLEMENTARY REFERENCES**

1. Chew J-L, Loh Y-H, Zhang W, Chen X, Tam W-L, Yeap L-S, Li P, Ang Y-S, Lim B, Robson P, Ng H-H: **Reciprocal Transcriptional Regulation of Pou5f1 and Sox2 via the Oct4/Sox2 Complex in Embryonic Stem Cells.** *Mol Cell Biol*

2005, **25:**6031-6046.

2. Lehnertz B, Ueda Y, Derijck AAHA, Braunschweig U, Perez-Burgos L, Kubicek

S, Chen T, Li E, Jenuwein T, Peters AHFM: **Suv39h-Mediated Histone H3**

**Lysine 9 Methylation Directs DNA Methylation to Major Satellite Repeats at**

**Pericentric Heterochromatin.** *Current Biology* 2003, **13:**1192-1200.
